# The New Pharmacopeia

**Published:** 1905-09

**Authors:** R. P. Daniel

**Affiliations:** San Antonio, Texas


**For Texas Medical Journal.**

### The New Pharmacopeia.

BY R. P. DANIEL, SAN ANTONIO, TEXAS.

*Texas Medical Journal, Austin, Texas:*

The eighth revision of the Pharmacopeia of the United States is now ready and is official. Many and some very important changes have been made in this most necessary of all books to the pharmacist, and it is highly important that every physician should know what these changes are.

Prof. Jos. P. Remington, Chairman of the Committee on Revision, assisted by an able corps of colaborators, has not only turned out the best work from every standpoint, but the cheapest; but it is cheap in price only. It is a handsome volume of 762 pages and sells for $2.50, in cloth binding. P. Blakiston & Son, Philadelphia, are distributors, and J. B. Lippincott & Co. are the publishers. It is worthy of a much more extended review than our space will permit. First, it is the most complete Pharmacopeia ever published in the United States. Nothing essential is left in doubt, but the very completeness of this arrangement makes it all the more necessary to bear in mind that every loop-hole of escape is closed in case of dereliction; for this work will be the legal standard in all States in case of legal proceeding regarding the dispensing of medicine, where identity and purity are concerned.

First of all, attention must be called to the change of strength in the tinctures, as shown in the following table, *especial attention being called to the first and last item of the list:*

| Name. | Present strength. Per cent. | Former strength. Per cent. |
|---|---|---|
| Tinct. Aconite | 10 | 35 |
| Tinct. Belladonna | 10 | 15 |
| Tinct. Benzoin Comp | 10 | 12½ |
| Tinct. Cannabis Indica | 10 | 15 |
| Tinct. Cantharides | 10 | 5 |
| Tinct. Capsicum | 10 | 5 |
| Tinct. Colchicum Seed | 10 | 15 |
| Tinct. Digitalis | 10 | 15 |
| Tinct. Gelsemium | 10 | 15 |
| Tinct. Hydrastis | 10 | 20 |
| Tinct. Hyoscyamus | 10 | 15 |
| Tinct. Lobelia | 10 | 20 |
| Tinct. Nux Vomica* | 10 | 10 |
| Tinct. Opium* | 10 | 10 |
| Tinct. Opium, Deodorized* | 10 | 10 |
| Tinct. Physostigma | 10 | 10 |
| Tinct. Sanguinaria | 10 | 10 |
| Tinct. Strophanthus | 10 | 5 |
| Tinct. Squill | 10 | 15 |
| Tinct. Stramonium | 10 | 15 |
| Tinct. Veratrum | 10 | 40 |

The following tinctures have been *doubled in strength:* Columba, Cardamon, Cinnamon, Quassia, Rhubarb, Serpentaria and Tolu. The following have been *reduced one-half in strength:* Gambir Compound (Catechu Comp.) and Tr. Kino.

Tinct. Sweet Orange Peel has been reduced from 50 per cent to 20 per cent.

The 10 per cent tinctures will be known as the *potent tinctures,* and some radical changes have been made to get all of the powerful tinctures brought to a single standard of 10 per cent. The physician and pharmacist must understand each other and be on their guard to prevent serious mistakes from these changes. I would suggest that the prescriber specify U. S. P., 1890, when any of the old tinctures are used, otherwise there is a chance that the patient might not get the dose intended.

These changes were made on the recommendation of the delegation which attended the conference in Brussels in September, 1902, which was composd of representatives of all civilized coun-

---

*Nux Vomica changed from 0.3 grams total alkaloids per 100 cubic centimeters to 0.1 gram strychnine to each 100 Cc.

Opium tinctures changed from 1.3 to 1.5 morphine to each 100 Cc. to 1.20 or 1.25 Gm. to each 100 Cc. of the tincture.

tries, who met for the purpose of making a standard of strength of all of the most common, powerful tinctures, and in accomplishing this they have practically made an International Pharmacopeia.

Other changes noted are: Carbolic Acid Ointment has been reduced from 5 per cent to 3 per cent, leaving room for still further reduction, as it is rarely necessary to use it full strength; white petrolatum being the base, which is an improvement. Ointment Sulphur is reduced from 30 per cent to 15 per cent. Mercurial Ointment is now made in strengths of 25 per cent and 50 per cent, instead of formerly one standard of 45 per cent. Solution of Iron and Ammonium Acetate, Basham's Mixture, has been doubled in strength, and, while we have not had time to try the new formula, it appears to be very similar to the old one, which, from a pharmaceutical standpoint, at least, was defective in appearance and keeping quality, because it was not made with a marked acid reaction. If this preparation is made so as to have a perceptible acid taste, it will keep for weeks, have an agreeable taste and a fine appearance.

Syrup of Ferrous Iodide has been reduced from 10 per cent to 5 per cent.

Solution of Chloride of Iron has been changed from 37.8 per cent to 29 per cent.

Solution of Tersulphate has been changed from 28.7 per cent to 36 per cent of $Fe_2(SO_4)_3$ by weight.

Effervescing Lithia Citrate reduced from 17 per cent to 5 per cent.

Effervescing Potassium Citrate reduced from 48 to 20 per cent.

Effervescing Caffein Citrate increased from 2 per cent to 4 per cent.

LIST OF ARTICLES ADDED TO THE PHARMACOPEIA.

Acetonum.
Acephenetidinum.
Acidum Camphoricum.
Acidum Hydroidicum.
Acid Hypophosphorosum.
Acidum Trichloraceticum.
Aconitina.
Adeps Lanae.
Aethylis Carbamas.
Aethylis Chloridum.
Ammonni Salicylas.
Aqua Hammamelidis.
Benzaldehydum.
Benzinum Purificatum.
Benzosulphinidum.
Berberis.
Bismuthi Subgallas.
Bismuthi Subsalicylas.
Bromoformum.
*Cataplasma Kaolini (Antiphlogistine).
Ceratum Resinae Compositum.
Chloralformamidum.
Cinnaldehydum.
Cocaina.
Codeinae Phosphas.
Codeinae Sulphas.
Colchicina.

*On the market under trade names.

Cresol.
Elixir Adjuvans.
Elixir Ferri et Quinia Phosphatum.
Emplastrum Adhaesivum.
Emulsum Olei Morrhuae.
Emulsum Olei cum Hypophos.
Emulsum Olei cum Terebinth.
Eugenol.
Extractum Malti.
Ext. Rhamni Pursianae.
Ext. Scopolae.
Ext. Stramonii.
Ext. Sumbul.
Fluidextractum Berberidis.
Fluidextractum Euonymi.
Fluidextractum Granati.
Fluidextractum Lobelia.
Fluidextractum Quercus.
Fluidextractum Quillajae.
Fluidextractum Rhamni Pursianae Aromat.
Fluidextractum Sanguinariae.
Fluidextractum Scillae.
Fluidextractum Scopolae.
Fluidextractum Stramonii.
Fluidextractum Sumbul.
Gambir.
Gelatinum.
Gelatinum Glycernatum.
Glandulae Supraenales Siccae.
Glandulae Thyroideae Siccae.
Glyceritum Ferri, Quiniae et.
Guaiacol.
Guaiacolis Carbonas.
Hamamelidis Cortex.
Hexamethylenamina.
Homatropinae Hydrobromidum.
Hydrastina.
Iodolum.
Kaolina.
*Liquor Antisepticus (Listerine).
Liquor Cresolic Compositus.
Liquor Formaldehydi.
Liquor Sodii Phosphatis Compositus.
Magnesii Sulphas Effervescens.
Maltum.
Mangani Hypophosphis.
Methylthioninae Hydrochloridum.
Oleatum Atropinae.
Oleatum Cocainae.
Opium Granulatum.
Paraffinum.
Pelletierinae Tannas.
Phenol Liquifactum.
Petrolatum Album.
Pilocarpinae Nitras.
Pilulae Laxitavae Compositae.
Pilulae Podophulli, Belladonnae et.
Pilulae Capsici.
Pulvis Acetanilidi Compsitus.
Quininae Salicylas.
Sabal.
Safrolum.
Scopola.
Scopolaminae Hydrobromidum.
Serum Antidiphthericum.
Sodii Arsenas Exsiccatus.
Sodii Carbonas Monohydrastus.
Sodii Citras.
Sodii Phosphas Effervescens.
Sodii Phosphas Exsiccatus.
Strontii Salicylas.
Strophanthinum.
Strychninae Phosphatum.
Sulphomethanum.
*Syrupus Hyphosphitum Compositus.
Talcum.
Talcum Purificatum.
*Thymolis Iodidum.
Tinctura Gambir Composita.
Tinctura Limonis Corticis.
Stramonii.
Trochi Gambir.
Unguentum Acidum Borici.
Unguentum Hydragyri Dilutum.
Unguentum Stramonii (leaf).
Unguentum Zinci Stearatis.
Vanilinum.
Vinum Cocae.
Zinci Phenolsuphonas.
Zinci Stearas.

*Doses.*—After each pharmacopeial article, which is to be used internally or hypodermically, the *average proximate dose* (but neither minimum or maximum) is given. The Committee on Revision wishes it distinctly understood that these doses are not obligatory on the physician, or as forbidding him to exceed them whenever in his judgment this seems advisable.

*Nomenclature.*—Many changes in names have been made, but solely for the purpose of insuring greater accuracy or safety in dispensing.

*Assay Process.*—An assay has been arranged for most of the

*On the market under trade names.

potent drugs whenever a process could be found which was practical for the use in an ordinary drug store. This is one of the features that shows greatest progress in pharmacy. All of the leading manufacturing houses have long ago adopted the system, and it is fitting that our pharmacopeia be fully their equal in this as well as other things.

*Purity.*—A limit of purity and strength of all pharmaceutical preparations is given in the following declaration of the Committee on Revision: *"The standards of purity and strength prescribed in the text of this Pharmacopeia are intended to apply to substances which are solely used for medicinal purposes, and when professedly bought, sold and dispensed as such."*

*The Metric System.*—While there is still opposition to the Metric System, it is undoubtedly the coming system for medical and pharmaceutical work, and has been adopted in the Pharmacopeia and is in our judgment superior to the make-shift of "parts by weight" used in the last revision, which could only be considered as the entering wedge for the metric system.

*New Articles.*—One hundred and seventeen new articles have been admitted (see table herewith), and 151 have been dismissed (list not given for lack of space). Of the new articles, twelve have been on the market under trade names for some time past. The patents on these preparations have either expired or will expire shortly, and only those admitted were of such nature that their identity and purity can be readily ascertained. The list is as follows:

Acetphenetidinum (Phenacetine).
Antipyrina (Antipyrine).
Benzosulphinidum (Saccharin).
Bromoformum (Bromoform).
Chloralformamidum (Chloralaid).
Guiacolis Carbonas (Duotal).
Hexamethylenamina (Urotropine).
Iodolum (Iodol).
Methylthioninæ Hydrochloridum (Methylene Blue).
Sulphonethylmethanum (Trional).
Sulphomethanum (Suphonal).
Thymolis Iodidum (Aristol).

The previous revision only contained acetanilid and salol, now changed to phenyl salicylate. Other notable new preparations are a few that have been before the public as proprietary preparations —Syrup of Hyphosphites Compound with Manganese, etc.

